# Prefrontal Control and Internet Addiction: A Theoretical Model and Review of Neuropsychological and Neuroimaging Findings

**DOI:** 10.3389/fnhum.2014.00375

**Published:** 2014-05-27

**Authors:** Matthias Brand, Kimberly S. Young, Christian Laier

**Affiliations:** ^1^Department of General Psychology: Cognition, University of Duisburg-Essen, Duisburg, Germany; ^2^Erwin L. Hahn Institute for Magnetic Resonance Imaging, Essen, Germany; ^3^Center for Internet Addiction, Russell J. Jandoli School of Journalism and Mass Communication, St. Bonaventure University, Olean, NY, USA

**Keywords:** Internet addiction, executive functions, cue-reactivity, craving, neuroimaging

## Abstract

Most people use the Internet as a functional tool to perform their personal goals in everyday-life such as making airline or hotel reservations. However, some individuals suffer from a loss of control over their Internet use resulting in personal distress, symptoms of psychological dependence, and diverse negative consequences. This phenomenon is often referred to as Internet addiction. Only Internet Gaming Disorder has been included in the appendix of the DSM-5, but it has already been argued that Internet addiction could also comprise problematic use of other applications with cybersex, online relations, shopping, and information search being Internet facets at risk for developing an addictive behavior. Neuropsychological investigations have pointed out that certain prefrontal functions in particular executive control functions are related to symptoms of Internet addiction, which is in line with recent theoretical models on the development and maintenance of the addictive use of the Internet. Control processes are particularly reduced when individuals with Internet addiction are confronted with Internet-related cues representing their first choice use. For example, processing Internet-related cues interferes with working memory performance and decision making. Consistent with this, results from functional neuroimaging and other neuropsychological studies demonstrate that cue-reactivity, craving, and decision making are important concepts for understanding Internet addiction. The findings on reductions in executive control are consistent with other behavioral addictions, such as pathological gambling. They also emphasize the classification of the phenomenon as an addiction, because there are also several similarities with findings in substance dependency. The neuropsychological and neuroimaging results have important clinical impact, as one therapy goal should enhance control over the Internet use by modifying specific cognitions and Internet use expectancies.

## Introduction

### General introduction and search methods

Most people use the Internet as a functional tool in everyday-life and many individuals cannot imagine living without the Internet in business or private life. The Internet provides multifariousness of possibilities for communication, entertainment, and dealing with everyday-life requirements (e.g., making restaurant reservations, searching for information, keeping updated with respect to political and society issues, etc.). With the growth of the Internet over the last two decades, the number of subjects experiencing massive negative consequences in their lives has also grown extensively. These persons experience a loss of control over their Internet use and report social problems as well as school and/or work difficulties (Young, 1998a; Beard and Wolf, 2001).

This contribution is a narrative review on Internet addiction and prefrontal control processes. It reflects the ideas and opinions of the authors based on their literature search and experiences. Nevertheless, we would like to briefly comment on the procedure we used to select the articles referred to in this review. We used two databases searching for suitable articles: PubMed and PsycInfo. The search was conducted using the terms: “Internet addiction,” “Compulsive Internet use,” and “Internet use disorder.” After a general overview over the found articles, each of the terms was combined with each of the terms “prefrontal cortex” or “executive functions” or “neuropsychology” or “control processes” or “decision making” or “neuroimaging” or “functional brain imaging” using the conjunction “AND.” Each term was required to be present in the “Title/Abstract” of the paper. Both searches were further limited by “English” as the publication language. We selected original research papers as well as review articles. We also used the function “related articles.” Given the limited space, we had to exclude several articles. We aimed at including both classical articles and very current studies. On the other hand, we also included some articles of other research areas (e.g., pathological gambling, substance dependency), whenever it seemed appropriate. In summary, following a systematic search for relevant articles, we selected the studies and reviews cited on the basis of a subjective impression. We thereby aimed at summarizing the most important views and findings on Internet addiction with a focus on the link between control processes and symptoms of Internet addiction. We also aimed at summarizing some very recent findings and ideas, which may be helpful for inspiring both future scientific studies and new therapeutic approaches.

### History of Internet addiction research, terminology, and symptoms

The first scientific description of a young man who developed severe psychosocial problems due to his excessive Internet use was done by Young (1996). It was followed by a growing number of other single- and multiple-case studies (e.g., Griffiths, 2000). Today, a relatively large literature exists on the phenomenology, the epidemiology for different countries, and co-morbidity of a problematic or pathological Internet use (see recent review by Spada, 2014). The prevalence rates reported in the last years have a wide variety from 0.8 in Italy to 26.7% in Hong Kong (see the excellent review by Kuss et al., 2013). Reasons for this extreme variance are most likely some cultural effects, but also the fact that until now, no standard assessment tool, no clearly defined cut-off scores, and even no fully accepted diagnostic criteria have been established (see exception for Internet Gaming disorder described below).

Although the clinical relevance is obvious and many clinicians see patients suffering from severe negative consequences due to an overuse of the Internet in general or certain Internet applications, the terminology used for this phenomenon and its classification are still under debate (Young, 1998b, 1999; Charlton and Danforth, 2007; Starcevic, 2013). Young (2004) argues that the criteria, which have been defined for pathological gambling and substance dependency should also be applied to Internet addiction. This is also in accordance with some other researchers, for example with the component model on addictive behaviors by Griffiths (2005). Nevertheless, there is a sum of different terms used in the scientific literature when referring to an overuse of the Internet, such as Internet addiction (Young, 1998b, 2004; Hansen, 2002; Chou et al., 2005; Widyanto and Griffiths, 2006; Young et al., 2011), compulsive Internet use (Meerkerk et al., 2006, 2009, 2010), Internet-related addictive behavior (Brenner, 1997), Internet-related problems (Widyanto et al., 2008), problematic Internet use (Caplan, 2002), and pathological Internet use (Davis, 2001). We prefer the term Internet addiction, since we see some important parallels between Internet addiction and other so-called behavioral addictions (e.g., Grant et al., 2013) and substance dependency (see also Griffiths, 2005; Meerkerk et al., 2009), which we will summarize in Sections “Neuropsychological Correlates of Internet Addiction” and “Neuroimaging Correlates of Internet Addiction.”

While there is great consensus about the multiple applications the Internet provides and which can be addictively used, such as gaming and gambling, pornography, social networking sites, shopping sites, and so on, only Internet Gaming Disorder has recently been included in the appendix of the DSM-5 (APA, 2013), making clear that more research is needed on this phenomenon to collect evidence for its clinical relevance and underlying mechanisms. The criteria proposed have significant similarities with the criteria used for diagnosing other forms of addiction and include:

- preoccupation with Internet games
- withdrawal symptoms of irritability, anxiety, or sadness
- development of tolerance
- unsuccessful attempts to control the behavior
- loss of interest in other activities
- continued excessive use despite knowledge of psychosocial problems
- deceiving others regarding the amount of time spent gaming
- use of this behavior to escape or relieve a negative mood
- jeopardizing/losing a significant relationship/job/educational opportunity

The APA has now focused on Internet gaming. We argue, however, that also other applications can be used addictively (Young et al., 1999; Meerkerk et al., 2006). Therefore, we summarize results of previous studies on Internet addiction in a broader way, although a great proportion of studies published so far concentrated on Internet gaming. Although not all criteria must be fulfilled, we would like to highlight one specific criterion, which seems very important and is most frequently fulfilled in patients suffering from Internet addiction. This criterion is: “Unsuccessful attempts to control the behavior” or to say it shorter: “Loss of control.” This criterion is also a factor frequently found when analyzing the factorial structure of questionnaires used to assess Internet addiction (Chang and Law, 2008; Korkeila et al., 2010; Widyanto et al., 2011; Lortie and Guitton, 2013; Pawlikowski et al., 2013). Consequently, the ability to control one’s own Internet use is an important factor preventing people from developing an Internet addiction. In turn, if an individual suffers from Internet addiction, one therapy goal must be to give the patient back the control over his/her Internet use. But why is it so difficult for some individuals to control the Internet use? One reason may be that Internet-related cues interfere with control processes mediated by the prefrontal cortex. We will summarize some recent findings from neuropsychological research emphasizing that in fact Internet-related stimuli interfere with decision making and other prefrontal functions, such as working memory and further executive functions. We will argue that reductions of prefrontal control processes play a major role in developing and maintaining an addictive use of the Internet.

Before we describe the role of control processes, we summarize recent models on Internet addiction, in order to make clear why specific cognitive processes may interact with other people’s characteristics, such as personality and psychopathological symptoms in the development and maintenance of Internet addiction in general or specific types of Internet addiction.

## Generalized and Specific Internet Addiction

Davis (2001) introduced a theoretical cognitive–behavioral model on pathological or problematic Internet use and differentiates between a generalized pathological Internet use, which we call generalized Internet addiction (GIA), and a specific pathological Internet use, for which we use the term specific Internet addiction (SIA). Davis argues that GIA is frequently linked to communicative applications of the Internet and that a lack of social support in real life and feelings of social isolation or loneliness are main factors contributing to the development of GIA. Maladaptive cognitions about the world in general and the own Internet use in particular may then intensify the overuse of the Internet to distract from problems and negative mood (see also Caplan, 2002, 2005). In contrast, for the overuse of certain Internet applications, for example, gambling sites or pornography, a specific individual predisposition is the main factor, Davis argues. Consequently, it is assumed that GIA is directly linked to the options the Internet itself provides, while SIA can also be developed outside the Internet, but is aggravated by the enormous functions offered by the Internet applications.

The model by Davis (2001) significantly inspired research on Internet addiction. However, neuropsychological mechanisms and – particularly – control processes mediated by executive functions and prefrontal brain areas have not been addressed directly. Additionally, we argue that reinforcing mechanisms conflict with control processes. Conditioning also plays an important role resulting in a strong relationship between Internet-related stimuli (or even computer-related stimuli) and positive or negative reinforcement. This conditioned relationship makes it increasingly harder for an individual to cognitively control the Internet use, even though negative consequences related to the Internet overuse are experienced in the long run. These kinds of conditioning processes are well-known for other forms of addiction and substance dependency (e.g., Robinson and Berridge, 2000, 2001; Everitt and Robbins, 2006; Robinson and Berridge, 2008; Loeber and Duka, 2009). We also argue that positive and negative reinforcement are differentially involved in the development and maintenance of GIA and SIA. Finally, we hypothesize that certain cognitions interact with control processes in developing and maintaining an addictive use of the Internet. Here, expectancies about what the Internet can provide and what a person may expect from using the Internet may be in a conflict with the individual’s expectancies about potential negative consequences in the short or the long run, which are associated with an Internet overuse.

**Figure 1:**
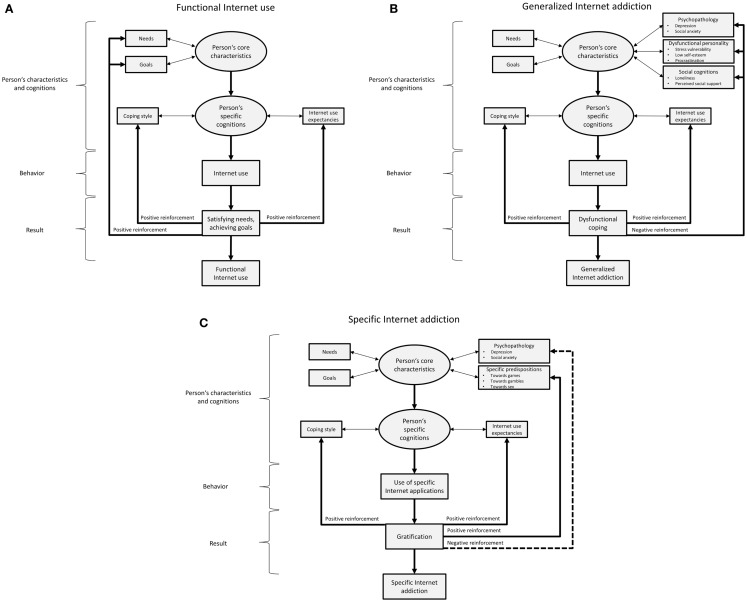
**The proposed model on the development and maintenance of generalized and specific Internet addiction**. **(A)** Demonstrates the proposed way of using the Internet as a tool for dealing with personal needs and goals in everyday-life. In **(B)**, the proposed mechanisms underlying generalized Internet addiction (GIA) are summarized. **(C)** Illustrates the proposed processes involved in specific Internet addiction (SIA), for example the addictive use of certain Internet applications, such as gaming, cybersex, communication, and so on. We argue that in both conditions, GIA and SIA, reductions in prefrontal control processes are related to the individuals’ loss of control over their Internet use. As outlined in Section “General Comments on Neuropsychological Research in Addiction,” control processes are related to higher-order cognitive functions. We believe that if an individual with GIA faces the situation that he/she is confronted with the possibility to go online (or to use a certain Internet application in an individual with SIA), these cues are so strong that the individual reacts relatively automatically with a wanting reaction. Cognitive control over this reaction is difficult if the expectancies that using the Internet would reduce craving and result in positive and/or negative reinforcement. We will summarize neuropsychological and neuroimaging findings on the link between executive control functions, cue-reactivity, and an addicted use of the Internet in Sections “Neuropsychological Correlates of Internet Addiction” and “Neuroimaging Correlates of Internet Addiction.”

Based on previous research and considering the theoretical arguments by Davis, we have recently developed a new model summarizing potential mechanisms, which contribute to the development to either GIA or SIA (see Figure 1). For the development and maintenance of GIA, we argue that the user has some needs and goals and that these can be satisfied by using certain Internet applications. We also assume that psychopathological symptoms, in particular depression and social anxiety (e.g., Whang et al., 2003; Yang et al., 2005) and dysfunctional personality facets, such as low self-efficacy, shyness, stress vulnerability, and procrastination tendencies (Whang et al., 2003; Chak and Leung, 2004; Caplan, 2007; Ebeling-Witte et al., 2007; Hardie and Tee, 2007; Thatcher et al., 2008; Kim and Davis, 2009) are predisposing factors for developing a GIA. In addition, social cognitions, such as perceived social isolation and a lack of social support offline are supposed to be related to GIA (Morahan-Martin and Schumacher, 2003; Caplan, 2005). These associations have already been well-documented in the literature. However, we believe that these predisposing characteristics act in concert with users’ specific cognitions. In particular, we argue that Internet use expectancies play an important role. These expectancies may involve anticipations of how the Internet can be helpful for distracting from problems or escaping from reality, or – more generally spoken – for reducing negative emotions. Those expectancies may also interact with the user’s general coping style (e.g., to tend toward substance abuse to distract from problems) and self-regulation capacities (Billieux and Van der Linden, 2012). When going online, the user receives reinforcement in terms of (dysfunctional) coping with negative feelings or problems in everyday-life. At the same time, the Internet use expectancies are positively reinforced, because the Internet acted as anticipated (e.g., reducing feelings of emotional or social loneliness). Given the strong reinforcing character of certain Internet applications, the cognitive control about the Internet use becomes more effortful. This should be particularly the case if Internet-related cues interfere with executive processes. We will go back to this topic in Sections “Neuropsychological Functions in Subjects with Internet Addiction” and “Functional Neuroimaging in Internet Addiction.”

Regarding the development and maintenance of an addictive use of specific Internet applications (SIA), we argue – consistent with previous research and in accordance with the model by Davis (2001) – that psychopathological symptoms are particularly involved (Brand et al., 2011; Kuss and Griffith, 2011; Pawlikowski and Brand, 2011; Laier et al., 2013a; Pawlikowski et al., 2014). We also hypothesize that specific person’s predispositions increase the probability that an individual receives gratification from the use of certain applications and overuses these applications again. One example for such a specific predisposition is a high sexual excitation (Cooper et al., 2000a,b; Bancroft and Vukadinovic, 2004; Salisbury, 2008; Kafka, 2010), which makes it more likely that an individual uses Internet pornography, because he/she anticipates sexual arousal and gratification (Meerkerk et al., 2006; Young, 2008). We believe that the expectancy that such Internet applications can satisfy certain desires increases the likelihood that these Internet applications are used frequently, as assumed in addictive behavior in general (Robinson and Berridge, 2000, 2003; Everitt and Robbins, 2006) and that the individual can develop a loss of control over his/her use of such applications. As a result, gratification is experienced and consequently the use of such applications and also the specific Internet use expectancies and the coping style are reinforced positively. This has already been shown, for example for cybersex addiction (Brand et al., 2011; Laier et al., 2013a) and is most likely also a mechanism for online gaming (e.g., Tychsen et al., 2006; Yee, 2006). The more general psychopathological tendencies (e.g., depression and social anxiety) are supposed to be negatively reinforced. This may be due to the fact that also specific Internet applications (e.g., Internet pornography) can be used to distract from problems in the real life or to avoid negative feelings, such as loneliness or social isolation. The main arguments of our model are summarized in Figure 1.

In both conditions (GIA and SIA), the loss of control over the use of the Internet in general or of specific applications is supposed to be the main consequence of the conditioning processes of Internet-related cues and positive and negative reinforcement. The question remains how these processes interact with higher-order cognitive functions. For example, what are the mechanisms behind the behavior to use the Internet again and again, although a person explicitly knows that he/she will experience negative consequences in the long run? Do they have a myopia for the future or is the reaction to the Internet-related stimuli so strong that they experience cue-reactivity and craving, as it is well-known from substance dependency (e.g., Grant et al., 1996; Anton, 1999; Childress et al., 1999; Tiffany and Conklin, 2000; Bonson et al., 2002; Brody et al., 2002, 2007; Franken, 2003; Dom et al., 2005; Heinz et al., 2008; Field et al., 2009)? We will focus on these neuropsychological mechanisms potentially contributing to the loss of control in the next sections.

## Neuropsychological Correlates of Internet Addiction

### General comments on neuropsychological research in addiction

**Figure 2:**
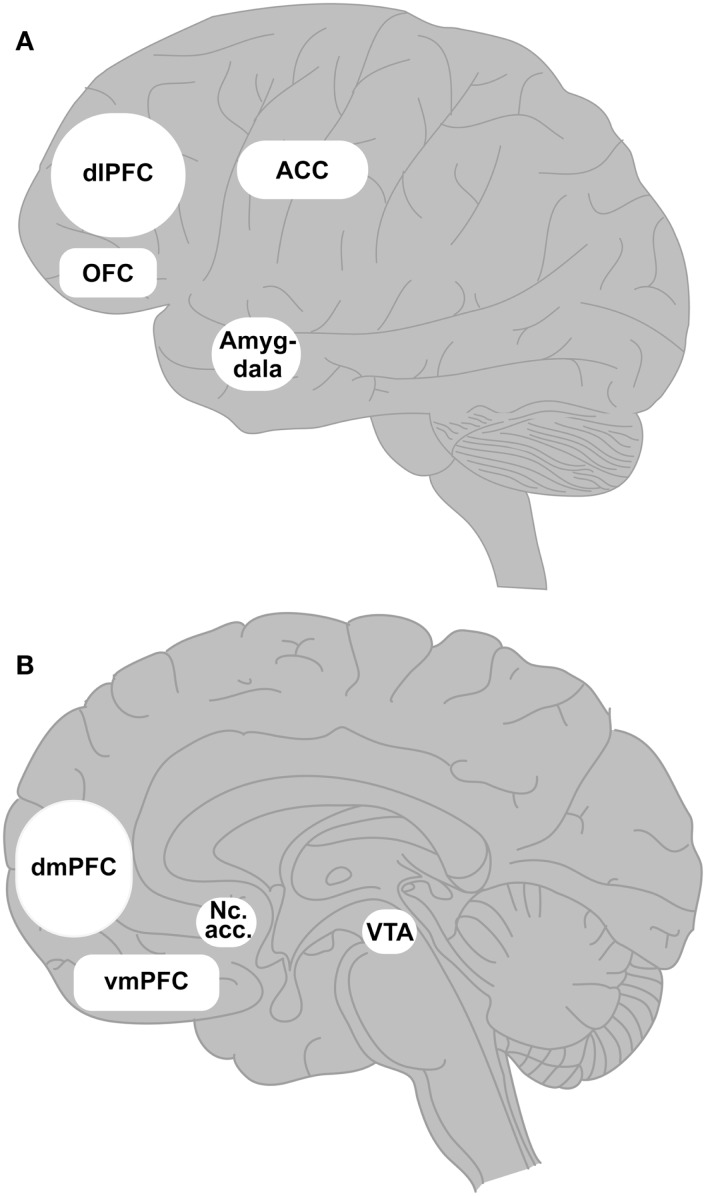
**The prefrontal cortex regions and associated brain structures most likely involved in development and maintenance of an addictive use of the Internet**. **(A)** Shows the lateral view of the brain including medial parts such as anterior cingulate gyrus and amygdala, and **(B)** illustrates the medio-sagittal view of the prefrontal cortex. These cortical and subcortical brain regions are main correlates of substance addiction and other behavioral additions. The dorsolateral prefrontal cortex (dlPFC) plays a crucial role in executive functions, cognitive control, and decision making under explicit conditions. It is connected to several basal ganglia, in particular to the nucleus caudatus and putamen via the so-called fronto-striatal loops. The orbitofrontal cortex (OFC) and the ventromedial prefrontal cortex (vmPFC) are critically linked to reward anticipation, emotion processing, and decision making under ambiguity. These structures are connected with limbic structures (amygdala) and the ventral striatum (nucleus accumbens, Nc. acc.) via the limbic part of frontal–striatal loops. The nucleus accumbens receives direct dopaminergic and indirect (via glutamate and GABA) projections from the ventral tegmental area (VTA) of the midbrain. The dorsomedial prefrontal cortex (dmPFC) and the anterior cingulate cortex (ACC) receive dopaminergic projections from the nucleus accumbens, and they are most likely involved in the so-called wanting component of craving. The anterior cingulate gyrus has also been discussed as being critical for conflict processing.

Cognitive control refers to the ability to control one’s own actions, behavior, and even thoughts and is a multifarious construct (Cools and D’Esposito, 2011). Although reductions in cognitive control are sometimes regarded as the main component of impulsivity, in neuropsychological research control mechanisms are ascribed to executive functions. Executive functions are control systems allowing us to regulate our behavior that is planned, goal oriented, flexible, and effective (Shallice and Burgess, 1996; Jurado and Rosselli, 2007; Anderson et al., 2008). These functions are strongly linked to parts of the prefrontal cortex, in particular the dorsolateral prefrontal cortex (e.g., Alvarez and Emory, 2006; Bari and Robbins, 2013; Yuan and Raz, 2014). The prefrontal cortex is connected to parts of the basal ganglia (e.g., Hoshi, 2013). For these connections, the term fronto-striatal loops is frequently used. Fronto-striatal loops include a more cognitive loop, which mainly connects the nucleus caudatus and putamen with the dorsolateral section of the prefrontal cortex (via the thalamus) and the limbic loop connecting limbic structures, such as the amygdala, and structures that are linked to motivational aspects of behavior, such as the nucleus accumbens, with the orbitofrontal and ventromedial part of the prefrontal brain area (Alexander and Crutcher, 1990). These parts of the brain are crucially involved in executive functions and other higher-order cognitions, but they are also main neural correlates of addictive behavior. Figure 2 summarizes these brain structures.

Before we focus on this issue in Section “Neuroimaging Correlates of Internet Addiction,” neuropsychological correlates of an addictive use of the Internet are summarized. In addiction research with a neuropsychological focus, executive functions, decision making, and attentional processes have been investigated extensively using traditional neuropsychological tasks, such as gambling tasks. These approaches have already been transferred to behavioral addictions, such as pathological gambling (e.g., Goudriaan et al., 2004; Brand et al., 2005b; Goudriaan et al., 2005, 2006; van Holst et al., 2010; Conversano et al., 2012) and compulsive buying (e.g., Black et al., 2012).

### Neuropsychological functions in subjects with Internet addiction

Over the last years, a sum of studies has also been published, which assessed general neuropsychological functions in individuals with either GIA or a certain SIA. Most of the studies, however, were done with excessive Internet gamers. One example is the study by Sun et al. (2009). They used the Iowa Gambling Task (Bechara et al., 2000), which had been used in many studies with different patient populations with neurological and psychiatric diseases including substance dependency and behavioral addictions before (cf. Dunn et al., 2006). This task assesses decision making under ambiguous conditions. Performing well on the task requires particularly learning from feedback. The excessive Internet users in the study by Sun et al. (2009) had problems in performing the Iowa Gambling Task, indicating decision-making deficits, which had frequently been linked to addictive behaviors (Bechara, 2005). In another study by Pawlikowski and Brand (2011), it was shown that excessive Internet gamers make more risky and disadvantageous choices, even when the rules for positive and negative consequences are explicitly explained, measured by the Game of Dice Task (Brand et al., 2005a). This result is consistent with findings in other samples with addiction, such as opiate dependency (Brand et al., 2008b), and pathological gambling (Brand et al., 2005b). Furthermore, performing the Dice Task well is linked to prefrontal integrity (Labudda et al., 2008) and executive functions (e.g., Brand et al., 2006; Brand et al., 2008a, 2009). Consequently, the results suggest that patients with Internet addiction may have reductions in prefrontal control and other executive functioning.

With respect to the ability to inhibit responses to certain stimuli, the individuals investigated by Sun et al. (2009) performed normally on a Go/No-Go Task, which measures response inhibition functions. This result on intact response inhibition is consistent with the findings by Dong et al. (2010) and also consistent with normal performance on the classical Stroop paradigm (see behavioral data in Dong et al., 2013b). However, in another study, Dong et al. (2011b) reported higher response errors in the incongruent condition of the Stroop paradigm in male Internet addicted individuals. In all these studies on inhibitory control, however, neutral versions of the Go/No-Go task or the Stroop paradigm have been used, meaning that all stimuli were unrelated to the Internet. One may hypothesize that individuals with Internet addiction react differently on stimuli, which explicitly show Internet-related content and have difficulty in inhibiting responses only to those stimuli, as it has been shown in substance-dependent individuals (e.g., Pike et al., 2013). This was reported by Zhou et al. (2012) using a shifting-task with Internet game-related cues. The authors argue that reductions in response inhibition and lower mental flexibility may be responsible for the maintenance of Internet gaming addiction.

Concentrating on other forms of Internet addiction, namely the excessive use of Internet pornography, which is also one of the main types of SIA (Meerkerk et al., 2006), beyond Internet gaming, first studies have used classical paradigms assessing cognitive functions and modified them in terms of including Internet pornographic pictures as stimuli. For example, Laier et al. (2014) used the Iowa Gambling Task, but included pornographic and neutral pictures on the card decks. One group of participants performed the task with pornographic pictures on the disadvantageous decks (A and B) and neutral pictures on the advantageous decks (C and D) and the other group performed the task with reversed picture-deck association (pornographic pictures on the advantageous decks C and D). The results demonstrated that the group performing the task with pornographic pictures on the disadvantageous decks had lower scores than the other group. This means that they continued selecting the cards from the decks with pornographic pictures, even though they received high losses. This effect was particularly observed in subjects who responded with a subjective craving reaction on the presentation of pornographic stimuli (in another paradigm, also included in the study). This finding is consistent with the results of another study by the same group of authors (Laier et al., 2013b), in which they reported lower working memory performance for pornographic stimuli than for positive, negative, and neutral pictures. The authors conclude that sexual arousal as reaction to Internet pornographic pictures interferes with cognitive functions.

We now argue that particularly cognitive control processes are affected when Internet addicted individuals are confronted with the addiction-related stimuli. However, this hypothesized mechanism needs further investigations for certain types SIA. Most importantly, this mechanism can be investigated best by using cognitive tasks, which include addiction-related stimuli and not with simple standard cognitive tasks.

## Neuroimaging Correlates of Internet Addiction

### General comments on neuroimaging research in the context of addiction

Most studies investigating neural correlates of Internet addiction with functional imaging techniques have been conducted with Internet gamers. These studies have revealed great similarities with brain circuits involved in the problematic behavior in substance-related addictions and pathological gambling, which will be discussed in the following sections. Two different approaches can be distinguished: functional activation studies as well as structural investigations and resting-state imaging including diffusion tensor imaging. The goal of both approaches is the same: a better understanding of the brain mechanisms involved in the excessive and addictive use of the Internet or certain Internet applications. The overall research questions are: does the brain change over time insofar that it learns to react on Internet cues specifically, and do these brain reactions determine the loss of control over the Internet use? From substance-dependency research, it is well-known that different brain areas are involved in the controlled and deliberative substance intake (e.g., with respect to alcohol) as compared to an uncontrolled and habitual use. In the first stages of drug-dependency development, frontal brain areas are particularly involved in the decision to consume a certain drug, motivated by its reinforcing effects (Goldstein and Volkow, 2002). As a result of classical and instrumental conditioning processes (Everitt and Robbins, 2006), the nucleus accumbens and parts of the dorsal striatum together with limbic and para-limbic regions (e.g., the orbitofrontal cortex) learn to habitually react on drug cues with craving and the dorsolateral prefrontal cortex, which is linked to higher-order cognitive functions, loses its regulatory influences (Bechara, 2005; Goldstein et al., 2009). This is most likely the consequence of changes in the dopaminergic reward system by frontal-guided changes of glutaminergic innervation of the nucleus accumbens and related brain areas (Kalivas and Volkow, 2005). In individuals with substance-dependency environmental factors, such as the presence of drug-related cues, lead to activations of the ventral striatum, the anterior cingulate cortex, and also mediofrontal cortex areas (Kühn and Gallinat, 2011; Schacht et al., 2013). These areas, but also the amygdala and the orbitofrontal cortex, are related to craving (Chase et al., 2011). In the next section, we will summarize previous neuroimaging findings on neural correlates of Internet addiction and will argue that the processes underlying substance dependency are also valid for Internet addiction.

### Functional neuroimaging in Internet addiction

Current studies on Internet addiction and in particular on Internet gaming addiction have applied neuroimaging methods to identify brain circuits involved in cue-reactivity and craving in those individuals who experience a loss of control over their Internet (games) use. A systematic review of those studies published in 2012 and earlier has been provided by Kuss and Griffiths (2012). They identified 18 studies, which used either functional magnetic resonance imaging (fMRI), positron emission tomography (PET), structural MRI or electroencephalography (EEG). When excluding the EEG studies (six studies summarized by Kuss and Griffith) and the two structural MRI studies, the systematic review concentrated on 10 studies with classical functional brain methods. We now applied the same search and inclusion criteria as documented in the review by Kuss and Griffiths (2012) and identified 13 studies (excluding EEG studies) published in peer-reviewed journals from January 2013 to end of January 2014. We here concentrate exemplarily on those earlier and current studies, which notably contribute to a better understanding of the link between prefrontal control processes and loss of control of the Internet use in individuals with Internet addiction.

One of the earliest studies on potential brain correlates of craving in subjects with Internet (gaming) addiction was reported by Ko et al. (2009). They studied excessive World-of-Warcraft (WoW) players (all participants played at least 30 h a week) with fMRI using a picture paradigm, which is comparable with those previously used in alcohol addiction research (e.g., Braus et al., 2001; Grüsser et al., 2004). The results were very similar to those reported in substance-dependent individuals (Schacht et al., 2013). The WoW players had, compared to the control group, stronger activations within the nucleus accumbens, the orbitofrontal cortex, and the caudate while watching WoW pictures. These activities were also correlated positively with subjective gaming urge. A comparable finding was reported by Sun et al. (2012), who also investigated excessive WoW players with a picture paradigm to induce craving. Here, activities in bilateral sections of the prefrontal cortex, in particular the dorsolateral prefrontal cortex, and the anterior cingulate cortex were positively correlated with subjective craving when watching WoW pictures. The results emphasize the view that the brain of Internet addicted individuals reacts with craving to the confrontation with Internet-related cues in the same way as the brain of substance-dependent individuals reacts on substance-related stimuli. Consistent with this, Han et al. (2011) found that the desire to play was positively related to activity in the right mediofrontal lobe and right parahippocampal gyrus even in healthy subjects, who were trained to play a certain video game for 10 days. Changes in prefrontal brain areas related to cue-reactivity and gaming urges in excessive players have also been reported in other previous studies (e.g., Han et al., 2010b; Ko et al., 2013a; Lorenz et al., 2013) and comparisons between cue-reactivity on gaming stimuli and substance dependency (e.g., tobacco) have been discussed (Ko et al., 2013b). Results illustrate similarities between Internet addiction and other addiction conditions with respect to underlying mechanisms of development, in particular conditioning processes (Robinson and Berridge, 2001, 2003; Thalemann et al., 2007). There is also some evidence for early functional brain adaptations in adolescent Internet users in frontal, temporal, and temporo-parietal–occipital junction area, as revealed by a ball-throwing paradigm (Kim et al., 2012). One first study linked cue-reactivity and craving with therapy success in subjects addicted to Internet games (Han et al., 2010a): at the first investigation with a picture paradigm and fMRI, the group of excessive StarCraft players (StarCraft is a real-time strategy video game), compared to volunteers with low StarCraft experiences, showed stronger activations in the dorsolateral prefrontal cortex, occipital areas, and left parahippocampal gyrus. Following a 6-week therapy with bupropion, which is frequently used in substance-dependence therapy, the craving reactions and playing time were reduced in the Internet gamers and the activity in the dorsolateral prefrontal cortex while watching StarCraft pictures were also decreased compared to the first fMRI investigation. Summarized, subjects with Internet addiction show craving reactions toward certain Internet-related cues on both subjective and neural level. Craving reactions are correlated with prefrontal brain changes, which are comparable to those reported for substance-dependent patients.

Also using fMRI, Dong et al. (2013b) investigated decision-making competence in individuals with Internet addiction (without specifying the type of Internet addiction). They used a card game with two options and manipulated the sequence of wins and losses, resulting in three conditions: continuous wins, continuous losses, and discontinuous wins and losses as control condition. Behaviorally, the individuals with Internet addiction needed longer for their decisions, in particular in the loss condition. Compared to the control subjects, the patients with Internet addiction had stronger brain activity in the inferior frontal gyrus, the anterior cingulate gyrus, and the insula in the win condition and stronger activity in the inferior frontal gyrus also in the loss condition. The posterior cingulate region and the caudate were less activated in patients with Internet addiction compared to the control group. The authors conclude that patients with Internet addiction have reductions in decision-making performance, because they need more endeavor to executive functions. In another publication with the same groups and tasks, the authors also reported a higher sensitivity for wins in comparison to losses in Internet addicted subjects (Dong et al., 2013a), which was accompanied by stronger activations in the inferior frontal gyrus and decreased activity in the posterior cingulate cortex in subjects with Internet addiction compared to the control group. These results fit with earlier investigations with the same guessing task (Dong et al., 2011a). Problems in making good decisions, meaning that individuals with Internet addiction continue playing games even though they are confronted with negative consequences, might be related to their problems in everyday-life (see also discussion in Pawlikowski and Brand, 2011). The argument of more endeavor in executive functions when being confronted with complex situations of decision making or when cognitive flexibility is required is confirmed by another fMRI study on cognitive flexibility of Internet addicted subjects (Dong et al., 2014). There is also first evidence for decreased error monitoring in subjects with Internet addiction, which is related to stronger activity in the anterior cingulate gyrus (Dong et al., 2013c), a region also known to be involved in cognitive control and conflict management (e.g., Botvinick et al., 2004). The results are consistent with another study on Internet addiction by Dong et al. (2012b), in which greater activity in the anterior (and also posterior) cingulate cortex was revealed for the interference condition of the Stroop paradigm.

Again, most studies used neutral stimuli when examining the neural correlates of cognitive functions in Internet addiction. Although these studies converge to the view that cognitive control processes are reduced in Internet addicted subjects, it would be important to investigate what happens in the brain of Internet addicts when being confronted with Internet-related stimuli. Given that individuals react with craving toward Internet-related cues (see literature review above), and that they obviously have some certain problems in executive control even in neutral situations, these executive and decision-making functions should be even worse when being in a situation, which offers Internet-related stimuli. This should be investigated in the future, because in daily life, the individuals are frequently confronted with the Internet and it would be clinically relevant to understand how the brain reacts toward those stimuli in interaction with reduced executive control functions.

### Structural and resting-state neuroimaging in Internet addiction

A study on both structural and functional neural correlates of Internet/computer gaming with a large sample (*N* = 154) adolescents reported higher gray matter volume in left ventral striatal region in frequent/excessive compared to infrequent players (Kühn et al., 2011). In the functional part of the study, activity in the region of the ventral striatum was higher in frequent compared to infrequent players in the loss condition of a monetary incentive delay task. The authors conclude that the volume changes in the left ventral striatal region may reflect changes in reward sensitivity linked to frequent playing of computer games. Gray matter density was also examined by Yuan et al. (2011). In a smaller sample (*N* = 18) of adolescents with Internet addiction, decreased gray matter volume was found in several prefrontal regions: the dorsolateral prefrontal cortex (bilaterally), the orbitofrontal cortex, and the supplementary motor area, as well as in posterior parts of the brain (cerebellum and the left rostral anterior cingulate cortex). The changes in the prefrontal areas were correlated with reported duration of the disorder. The authors conclude that these brain changes may be responsible for an impairment of cognitive control in subjects with Internet addiction and that these changes have some important similarities with those observed in substance dependency. Reductions in gray matter density were also found in the left anterior and posterior cingulate cortex, as well as in the insula (Zhou et al., 2011) and in the orbitofrontal cortex (Hong et al., 2013a; Yuan et al., 2013). The changes in the orbitofrontal region were correlated with performance in the Stroop paradigm (Yuan et al., 2013), indicating functional reductions in prefrontal control processes. Gray matter reductions in the (right) orbitofrontal cortex in individuals with SIA for games, in addition also in the insula (bilaterally), and the right supplementary motor area were reported by Weng et al. (2013). Interestingly, the volume of the orbitofrontal cortex was correlated with the scores in the Internet Addiction Test (Young, 1998a), measuring symptom severity.

In addition to gray matter, abnormalities in patients with Internet addiction, functional connectivity shows some changes. These connectivity alterations fit well, at least partially, with the structural changes. For example, Lin et al. (2012) found lower fractional anisotropy in large parts of the brain of individuals with Internet addiction including the orbitofrontal cortex. Further changes in fractional anisotropy were found in the white matter of the parahippocampal gyrus (Yuan et al., 2011), bilateral frontal lobe white matter (Weng et al., 2013), and both internal (Yuan et al., 2011) and external capsule (Weng et al., 2013). Also, reductions in functional connectivity (using resting-state fMRI) were found in the right inferior temporal gyrus, bilateral parietal cortex and posterior cingulate cortex, and connectivity between the posterior cingulate gyrus and right precuneus, parts of the thalamus, caudate, ventral striatum, supplementary motor area, and lingual gyrus was correlated with severity of the problematic behavior in Internet gamers (Ding et al., 2013). However, in another study by Dong et al. (2012a), using diffusion tensor imaging, increased connectivity between several brain areas in patients with Internet addiction for games were reported, including thalamus and posterior cingulate cortex. The fractional anisotropy in the internal capsule was also correlated with the duration of the addictive behavior (Yuan et al., 2011). Reduced connectivity was also found between prefrontal and subcortical as well as parietal and subcortical structures, in particular with the putamen (Hong et al., 2013b). There are some references for changes in regional homogeneity with both increased homogeneity in middle frontal and parietal gyri (and further regions of brainstem and cerebellum) and decreased homogeneity in certain temporal, parietal, and occipital areas in individuals with Internet gaming addiction (Dong et al., 2012c).

Another line of arguments for the involvement of cue-reactivity and craving, which might interfere with cognitive control over the Internet use, comes from studies investigating the dopamine system in patients with Internet addiction. Although these studies are preliminary given, for example, very small samples sizes and their results have to be treated with caution: there are some first hints that the dopamine system is altered in Internet addicted individuals. One example is a SPECT study (Hou et al., 2012) showing that the level of dopamine transporter expression in the striatum is decreased in individuals with Internet addiction. This finding is consistent with the results of a study with raclopride PET (Kim et al., 2011), in which a reduced availability of dopamine 2 receptors in the striatum was found in Internet addicts (see also the review by Jovic and Ðinđić, 2011).

Although this is speculative so far, changes in dopaminergic functioning may – at least partly – explain the loss of control over the Internet use in individuals with Internet addiction. This assumption fits well with recent models on the development of addictive behavior in general, as suggested by Robinson and Berridge (2008), as already mentioned. Given that the parts of the prefrontal cortex involved in cognitive control, in particular the dorsolateral prefrontal cortex (see Figure 2) receives dopaminergic projections from the basal ganglia and the nucleus accumbens, functional changes in these structures can also reduce the integrity of executive control (Cools and D’Esposito, 2011). Given that the basal ganglia are inter-connected with each other and the thalamus by projections that include other neurotransmitter systems, in particular glutamate and GABA, changes in the dopaminergic system may also cause more global dysfunctions of the fronto-striatal loops, including both the cognitive and the limbic loop (Alexander and Crutcher, 1990). We have commented on the link between fronto-striatal loops and executive control functions in Section “Neuropsychological Correlates of Internet Addiction.” Considering the preliminary results on dopaminergic alterations in Internet addicted individuals, we argue that changes in this and other basal ganglia neurotransmitter systems are related to the loss of control over the Internet use by functional changes of prefrontal integrity.

Beyond the investigations of the dopamine system, further studies have addressed resting-state brain functionality in patients with Internet addiction. Using 18-FDG-PET, measuring glucose metabolism in the brain, Park et al. (2010) demonstrated that excessive Internet gamers had increased glucose metabolism in the region of the (right) orbitofrontal cortex, and also in parts of the basal ganglia (left caudate, insula), while posterior regions (e.g., parietal and occipital areas) showed decreased metabolism.

In summary, there are some first evidences for structural and resting-state brain changes in individuals with Internet addiction. These include both gray and white matter changes in the prefrontal brain areas and additional brain regions. There are also first evidences for changes in the dopaminergic system, which might be related to reinforcement processing and craving. Given that most studies were done with rather small samples, with one exception only (Kühn et al., 2011), and no consistent or systematic differentiation between different types of Internet addiction and between adolescent versus adult patients, the results must be treated with caution.

## Summary and Clinical Implications

In summary, neuropsychological and neuroimaging research on excessive and addictive use of the Internet is a rapidly growing scientific field, which has revealed a sum of very interesting results. These results have both scientific and clinical impact and help to better understand the neurobiological basis of Internet addiction. The results converge to the view that an addictive use of the Internet is linked to functional brain changes involving parts of the prefrontal cortex, accompanied by changes in other cortical (e.g., temporal) and subcortical (e.g., ventral striatum) regions. Additionally, there are some hints for structural brain changes, which also involve parts of the prefrontal cortex. The functional changes in prefrontal and striatal areas are primarily observable when individuals with Internet addiction perform certain tasks, in particular those measuring executive functions and cue-reactivity. These results, together with those emerging from neuropsychological studies, suggest that prefrontal control processes are reduced in individuals who are addicted to the Internet and may be related to the patients’ loss of control over their Internet use. However, there are some limitations of the research findings existing so far. First, as already mentioned, the combination of assessing higher-order cognitive functions and the confrontation with Internet-related stimuli should be investigated more extensively. Second, more studies on different types of Internet addiction (i.e., different specific forms, such as gaming, communication, pornography) are needed to better understand common and specific neuropsychological and neural correlates of Internet addiction (GIA and certain types of SIA). Third, the age of participants has not been addressed systematically. While some studies were conducted on adolescents, other results were obtained from adult participants, and it is hard to compare the neural correlates of Internet addiction across different age groups. Fourth, little is known about gender as a further variable potentially influencing the underlying mechanisms of GIA and different types of SIA. However, most of the previous studies were done with male participants. Fifth, most of the neuroimaging studies were conducted in Asia. Although these studies have been excellently performed and are very influential in the field, some cultural effects on the phenomenon of Internet addiction cannot be excluded. Consequently, we need more studies on neuropsychological and neuroimaging correlates of an addictive Internet use in different countries using certain populations, including male and female participants of different age groups and with certain types of Internet addiction to systematically address and better understand this clinical phenomenon.

Assuming that the current results of reduced prefrontal control in Internet addicted individuals will be confirmed by further samples, we here discuss the potential impact on treatment procedures. The first treatment model for Internet addiction was introduced by Young (2011), which has been named cognitive–behavioral therapy for Internet addiction (CBT-IA). Cognitive–behavioral therapy is the method of choice (Cash et al., 2012; Winkler et al., 2013), although the number of empirical studies on treatment outcome is still limited (Young, 2013), as it is the case for other behavioral addictions (Grant et al., 2013). Within CBT-IA model proposed by Young (2011), individual characteristics as well as specific cognitions have been hypothesized to be key elements, which should be addressed in the therapy. CBT-IA consists of three phases, in which instantly Internet behavior is monitored in accordance to its incidental situational, emotional, and cognitive conditions as well as with its subsequent positive and negative reinforcing effects to identify cognitive assumptions and distortions about one’s own self, Internet use, situational triggers, and high-risk situations. In the second phase, cognitive biases about one’s own self and the Internet as well as denial about treatment is proposed to be analyzed and treated by methods of cognitive restructuring and reframing. In the third phase of treatment, personal, social, psychiatric, and occupational issues related to the development and maintenance of Internet addiction need to be understood and changed. The efficacy of all three treatment phases depends on prefrontal processes, in particular executive functions, such as planning, monitoring, self-reflection, cognitive flexibility, and working memory.

With respect to the proposed model on development and maintenance of GIA and SIA (Figure 1), control processes and executive functions may significantly influence the person’s cognitions, in particular coping style and Internet use expectancies. If a client has reduced prefrontal control processes, in particular in situations in which he/she is confronted with Internet-related cues, he/she may have difficulties in developing other coping strategies to deal with daily hassles than turning to the Internet. The reinforcement that is experienced when using the Internet may then strengthen the Internet use expectancies, which in turn may result in ignoring other ways to cope with negative mood. The client may focus his/her view on the world and the own cognitions on Internet-related issues and these cognitions are permanently reinforced (both positively and negatively) by using the Internet. Reduced prefrontal control processes may result in a restricted and cramped perception of situational features and ways to deal with everyday-life requirements. It is then even harder for the therapist to convey control mechanisms to the client, if prefrontal control processes are reduced. Monitoring and controlling situational triggers, which are fundamental ingredients in getting back the control over the Internet use, also rely on prefrontal control processes. We therefore argue that in the context of clinical treatment it is important to assess the client’s cognitive functions, in particular executive functions, before working with the client on her/his specific Internet-related cognitions. This is speculative, because no empirical study on neurocognitive functions as predictors of therapy outcome exists, so far. However, we argue that including neuropsychological training with a focus on general and Internet specific control processes should result in an even better outcome.

All the findings and clinical implications discussed here have several similarities with other forms of addictive behaviors. They are consistent with neurobiological and psychological models of addition (Robinson and Berridge, 2003; Everitt and Robbins, 2006) and with neuropsychological and neuroimaging findings in substance dependency and other forms of behavioral additions (Grant et al., 2006; van Holst et al., 2010). They should inspire incorporating neurobiological findings into treatment designs for Internet addiction, as it has been proposed for other forms of behavioral addictions (Potenza et al., 2013). Most of the current articles on neuropsychological and neuroimaging correlates of Internet addiction conclude that this clinically relevant disorder should be classified as a behavioral addiction. We agree with this conclusion and hope that this review will inspire future research on neuropsychological and neurobiological mechanisms of the development and maintenance of an addictive use of the Internet in general and certain Internet applications in specific, as well as on predictors for treatment efficacy.

## Author Contributions

Matthias Brand wrote the first draft of the paper, supervised the preparation of the manuscript, contributed intellectual and practical work to the manuscript, and revised the text. Kimberly S. Young edited the draft, revised it critically, and contributed intellectually and practically to the manuscript. Christian Laier contributed particularly to the theoretical part of the manuscript and revised the manuscript. All authors finally approved the manuscript. All authors are accountable for all aspects of the work.

## Conflict of Interest Statement

The authors declare that the research was conducted in the absence of any commercial or financial relationships that could be construed as a potential conflict of interest.
